# Severe Human Lassa Fever Is Characterized by Nonspecific T-Cell Activation and Lymphocyte Homing to Inflamed Tissues

**DOI:** 10.1128/JVI.01367-20

**Published:** 2020-10-14

**Authors:** Julia R. Port, David M. Wozniak, Lisa Oestereich, Elisa Pallasch, Beate Becker-Ziaja, Jonas Müller, Monika Rottstegge, Catherine Olal, Sergio Gómez-Medina, Jennifer Oyakhliome, Yemisi Ighodalo, Emmanuel Omomoh, Thomas Olokor, Donatus I. Adomeh, Danny Asogun, Ephraim Ogbani-Emovon, Kristin Hartmann, Susanne Krasemann, Emily V. Nelson, Beatriz Escudero-Pérez, Anita K. McElroy, Stephan Günther, César Muñoz-Fontela

**Affiliations:** a Bernhard Nocht Institute for Tropical Medicine, Hamburg, Germany; b German Center for Infection Research (DZIF), Partner Site Hamburg, Hamburg, Germany; c Robert Koch Institute, Berlin, Germany; d Institute of Lassa Fever Research and Control, Irrua Specialist Teaching Hospital, Irrua, Edo State, Nigeria; e Institute of Neuropathology, University Medical Center Hamburg-Eppendorf, Hamburg, Germany; f Department of Pediatrics, University of Pittsburgh, Pittsburgh, Pennsylvania, USA; St. Jude Children's Research Hospital

**Keywords:** Lassa virus, T cells, T-cell homing, pathogenesis, host response, Lassa fever, viral hemorrhagic fever

## Abstract

Lassa fever (LF) is a zoonotic viral hemorrhagic fever caused by Lassa virus (LASV), which is endemic to West African countries. Previous studies have suggested an important role for T-cell-mediated immunopathology in LF pathogenesis, but the mechanisms by which T cells influence disease severity and outcome are not well understood. Here, we present a multiparametric analysis of clinical immunology data collected during the 2017–2018 Lassa fever outbreak in Nigeria. During the acute phase of LF, we observed robust activation of the polyclonal T-cell repertoire, which included LASV-specific and antigenically unrelated T cells. However, severe and fatal LF cases were characterized by poor LASV-specific effector T-cell responses. Severe LF was also characterized by the presence of circulating T cells with homing capacity to inflamed tissues, including the gut mucosa. These findings in LF patients were recapitulated in a mouse model of LASV infection, in which mucosal exposure resulted in remarkably high lethality compared to skin exposure. Taken together, our findings indicate that poor LASV-specific T-cell responses and activation of nonspecific T cells with homing capacity to inflamed tissues are associated with severe LF.

**IMPORTANCE** Lassa fever may cause severe disease in humans, in particular in areas of endemicity like Sierra Leone and Nigeria. Despite its public health importance, the pathophysiology of Lassa fever in humans is poorly understood. Here, we present clinical immunology data obtained in the field during the 2018 Lassa fever outbreak in Nigeria indicating that severe Lassa fever is associated with activation of T cells antigenically unrelated to Lassa virus and poor Lassa virus-specific effector T-cell responses. Mechanistically, we show that these bystander T cells express defined tissue homing signatures that suggest their recruitment to inflamed tissues and a putative role of these T cells in immunopathology. These findings open a window of opportunity to consider T-cell targeting as a potential postexposure therapeutic strategy against severe Lassa fever, a hypothesis that could be tested in relevant animal models, such as nonhuman primates.

## Abstract (precis)

Lassa fever may cause severe disease in humans, in particular in areas of endemicity like Sierra Leone and Nigeria. Despite its public health importance, the pathophysiology of Lassa fever in humans is poorly understood. Here, we present clinical immunology data obtained in the field during the 2018 Lassa fever outbreak in Nigeria indicating that severe Lassa fever is associated with activation of T cells antigenically unrelated to Lassa virus and poor Lassa virus-specific effector T-cell responses. Mechanistically, we show that these bystander T cells express defined tissue homing signatures that suggest their recruitment to inflamed tissues and a putative role of these T cells in immunopathology. These findings open a window of opportunity to consider T-cell targeting as a potential postexposure therapeutic strategy against severe Lassa fever, a hypothesis that could be tested in relevant animal models, such as nonhuman primates.

## INTRODUCTION

Lassa fever (LF) is a severe viral disease endemic to West African countries, such as Nigeria, Guinea, Liberia, and Sierra Leone. The causative agent is Lassa virus (LASV) (species *Lassa mammarenavirus*, family *Arenaviridae*), a single-stranded bisegmented RNA virus. LF is a zoonotic disease, and seasonal outbreaks are caused by multiple spillover events from the virus’s rodent host, the multimammate rat *Mastomys natalensis*, into humans (1). In hyperendemic regions like southern Nigeria and Sierra Leone, LASV may cause up to 300,000 estimated LF cases and 5,000 deaths annually (1, 2). Although there are limited epidemiological data on LASV transmission, human infection is thought to occur mainly through contact with rodent excreta, which may involve consumption of contaminated food, inhalation of particles containing infectious virus, and skin contact with infected-rodent body fluids (2, 3). Contact with infectious body fluids is also the main route of human-to-human transmission either in the household or during nosocomial outbreaks (4).

An LF outbreak is defined as a significant increase of cases over the baseline number of cases that occur during the regular Lassa season. For reasons that are not well understood, outbreaks take place in areas of endemicity at random intervals. In 2018, the Nigerian LF season was characterized by the largest-ever-recorded number of cases in the country (5). Our laboratory, which is located at the Nigerian reference center for Lassa fever at the Irrua Specialist Teaching Hospital (ISTH), was engaged in outbreak control, diagnostics, and patient treatment. In this context, we set up an operational research protocol to better understand host factors driving disease severity.

In a previous study, we demonstrated that depleting T cells during the course of LF in a mouse model was sufficient to rescue mice from death (6), and severe LF disease was achieved in a mouse model expressing a human HLA-A2 transgene (7). However, these findings were restricted to infection models and their relevance in humans was not known. There is substantial evidence that in humans, as well as nonhuman primates, early T-cell activation is correlated with recovery and viral clearance (8, 9). Furthermore, fatal LF has been associated with lymphopenia in previous studies (10). However, studies on human immunology during acute disease are rare and the overall role of T cells in Lassa fever is poorly understood. During viral infections, there are several mechanisms by which T cells can influence pathogenesis, including activation of bystander T cells, activation of memory T cells due to latent virus reactivation, production of proinflammatory cytokines, and homing into inflamed tissues where T cells can directly cause tissue damage by killing infected target cells (11, 12).

Bystander activation of T cells independent of T-cell receptor (TCR) engagement, as well as their reactivation by latent virus infection, has been associated with exacerbation of pathogenesis in certain infections, including Lyme disease, leishmaniasis, HIV, dengue fever, and Ebola virus disease (13–15). Antigenically unrelated T-cell activation during acute infection occurs as a consequence of reactivation of latent virus infections, as well as through the effect of an inflammatory milieu, where antigen-independent T cells become effector T cells due to cytokine stimulation or through engagement of pattern-recognition receptors (PRRs) (11, 13). Massive recruitment of both virus-specific and -nonspecific T cells to skin and gut has been previously correlated with the immunopathology caused by infectious diseases like dengue, rotavirus, and herpesvirus infection (16–18). An additional consequence of nonspecific T-cell activation is the initiation of anti-inflammatory mechanisms, upregulation of T-cell immune checkpoints, and/or induction of T-cell anergy, which may cause an overall reduction of pathogen-specific T-cell responses (12, 19). However, the putative role of nonspecific T-cell activation in LF pathogenesis has not been yet investigated.

Both antigen-specific and -nonspecific T cells can be recruited into inflamed tissues via the expression of defined “homing” markers, such as cutaneous lymphocyte antigen (CLA) or lymphocyte Peyer’s patch adhesion molecule 1 [LPAM-1, or alpha(4)beta(7) integrin], which bind E-selectin and mucosal addressin cell adhesion molecule (MadCAM)-expressing cells, respectively (20–22), and thereby drive the recruitment of T cells to the skin and gut mucosa. Thus, through their expression of tissue-specific homing molecules, activated T cells are able to leave the blood and lymphatic systems and migrate into specific organ tissues. Consequently, infectious diseases with one primary route of transmission or strong tissue tropism are characterized by the presence of peripheral blood T cells with defined T-cell homing signatures (17, 21, 23). Thus, understanding T-cell homing patterns in LF could provide insight into migratory patterns of T cells during the acute phase of disease and their possible implications in immunity and immunopathology.

Here, we analyzed T-cell responses from patients diagnosed with LF during the 2017–2018 Lassa fever outbreak in Nigeria and showed that antigenically unrelated T-cell activation and poor LASV-specific effector T-cell responses were associated with severe disease. Moreover, severe LF was characterized by the presence of T cells with homing signatures to mucosal tissues. These findings suggest that recruitment to inflamed tissues of virus-specific and -nonspecific T cells with poor capacity for virus clearance may be an important component of LF pathophysiology.

## RESULTS

### Cohort description and clinical manifestations of LF.

**FIG 1:**
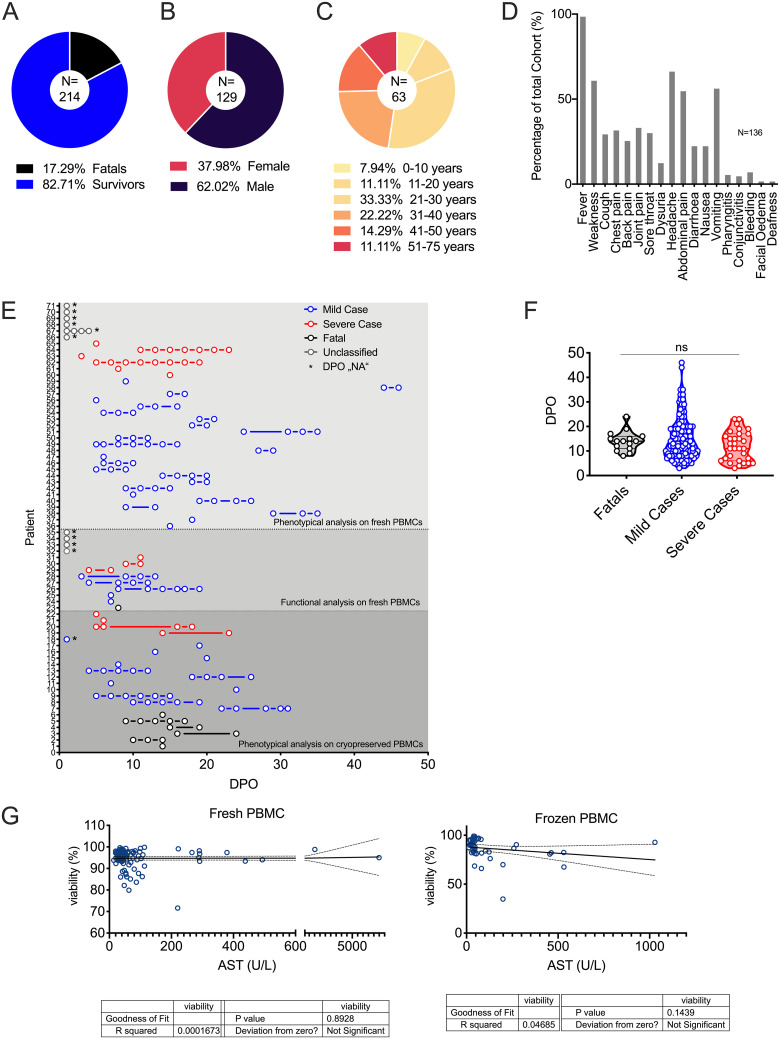
Patient demographics and clinical symptoms. (A to C) Outcome (*n* = 214) (A), gender (*n* = 129) (B), and age (*n* = 63) (C) distributions of patients admitted to the Lassa ward during the study period. Distributions of patients in population are shown as the percentages of the total cohort. Pie chart colors are described in keys below. (D) Symptoms of patients (*n* = 136) admitted to the Lassa ward. (E) PBMCs in whole-blood samples were analyzed in the field for 36 patients for phenotypical assessment and for 13 patients for assessment of the T-cell response, and for 22 acute patients, cryopreserved samples were analyzed phenotypically. The graph depicts patients and dates of sample collection according to the self-reported days postonset (DPOs). NA, not answered. (F) DPOs of patients at the time of sample collection were compared between patient groups with different outcomes. Violin plots depict individual samples, sample distributions, median values, and quartiles. ns, not significant (Kruskal-Wallis, followed by Dunn’s). (G) Viability of all fresh and frozen PBMC samples was assessed in relation to the corresponding aspartate transaminase (AST) serum levels (U/liter). Panels depict results for individual samples (fresh and frozen) and linear regression lines with 95% confidence intervals (dashed).

The LF cases included in this study were admitted to the Lassa ward at ISTH in Edo state, Nigeria, between January 2017 and June 2018. In total, *n* = 214 patients were diagnosed as positive for LF via real-time PCR (RT-PCR) and enrolled in our study, out of which 17% succumbed to the disease (Fig. 1A). Most of the enrolled patients came from the neighboring states of Edo and Ondo in southwestern Nigeria. Demographically, 62% of patients were male and 33% of the patients were between 21 and 30 years of age (Fig. 1B and C). Upon admission, all patients had fever and more than 50% reported weakness, headache, and gastrointestinal manifestations like abdominal pain and vomiting (Fig. 1D). Samples were collected from patients longitudinally during their stay in the Lassa ward, coincident with the acute phase of LF. An initial functional analysis of the T-cell response during acute LF was performed at ISTH (*n* = 13 patients), as well as a first assessment of T-cell phenotype markers (*n* = 36 patients). Further multiparametric assessment of T-cell phenotypes was performed on samples from *n* = 22 patients that were cryopreserved and shipped to the biosafety level 4 (BSL-4) laboratory at the Bernhard Nocht Institute for Tropical Medicine in Hamburg, Germany (Fig. 1E). Patients were classified as fatalities or survivors based on outcome. Among survivors, patients were classified retrospectively as severe cases if their levels of serum aspartate aminotransferase (AST) were higher than 300 U/liter at any point during disease, while cases were defined as mild if AST levels were lower than 300 U/liter throughout the course of LF. Upon admission, patients provided self-reported days post-symptom onset (DPO) estimates, which were comparable across mild, severe, and fatal cases (Fig. 1F). For flow cytometry analysis, sample viability was assessed in relation to corresponding serum AST levels, a biomarker of disease severity (Fig. 1G).

### Acute Lassa fever induces nonspecific T-cell activation.

**FIG 2:**
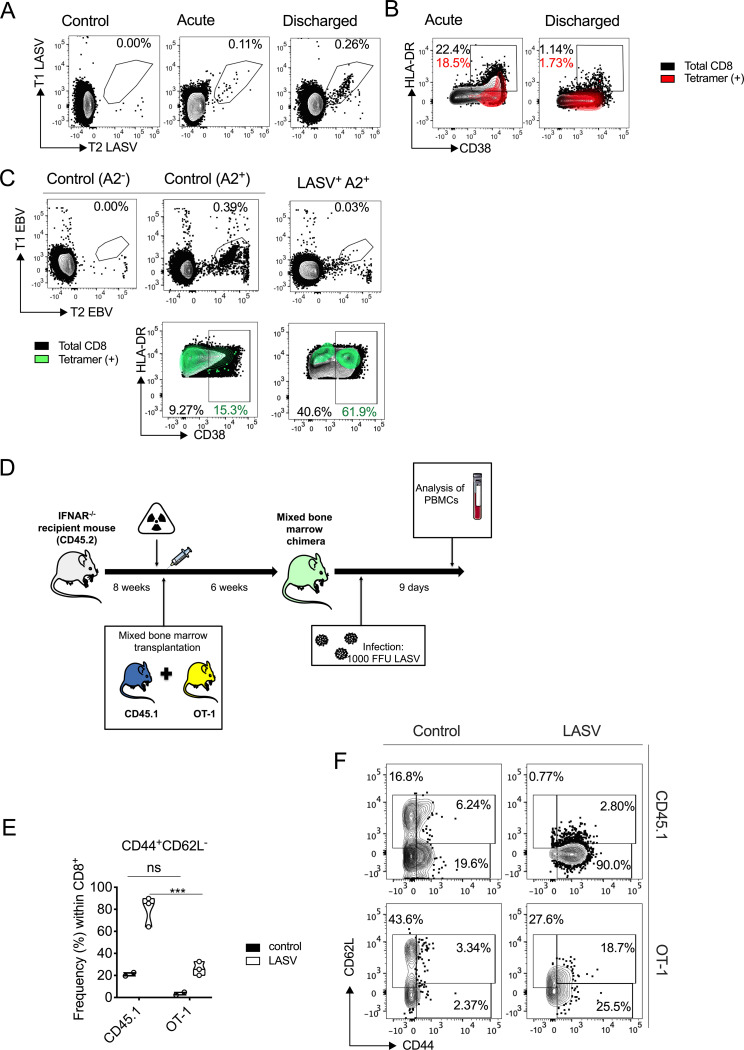
T-cell activation during acute Lassa fever (LF). (A) HLA-A2 tetramers labeled with two fluorophores (T1 and T2, respectively) were utilized to track epitope-specific CD8 T cells during acute LF. The plots show results for pooled (concatenated) samples from LF patients (*n* = 3) collected during the acute phase of LF or after discharge. (B) Overlay plots showing Lassa virus (LASV) epitope-specific CD8 T cells (red) plotted over the total activated (CD38^+^ HLA-DR^+^) population. (C) Epstein-Barr virus (EBV)-specific CD8 T cells were identified in acute LF patients through the use of tetramers labeled with two fluorophores (T1 EBV and T2 EBV). Plots show tetramer staining in negative control (LASV^−^ A2^−^) and uninfected control (LASV^−^ A2^+^) and a representative acute HLA-A2^+^ LF patient (*n* = 2) (LASV^+^ A2^+^). Bottom plots show overlay of EBV-specific CD8 T cells over total activated CD8 T cells. (D) Mixed CD45.1 (WT) and OT-I bone marrow chimeras were generated and infected intranasally with 1,000 focus-forming units (FFU) LASV as depicted in the schematic. IFNAR, IFN-α/β receptor. (E) Frequencies (%) of effector cells (CD44^+^ CD62L^−^) are shown for CD8 T cells isolated from mouse peripheral blood mononuclear cells (PBMCs) 9 days after infection (LASV) compared to the CD8 T cells from PBMCs of uninfected mice (control) for both CD45.1 (WT) and OT-1 cells. Violin plots depict individual samples, sample distributions, median values, and quartiles. Statistical significance was determined by 2-way analysis of variance (ANOVA) followed by Sidak’s multiple-comparison test, and significance levels are presented as follows: ns, *P* > 0.05 (not significant); *, *P* ≤ 0.05; **, *P* ≤ 0.01; ***, *P* ≤ 0.001; ****, *P* ≤ 0.0001. (F) Representative plots are shown depicting effector CD8 T cells (CD44^+^ CD62L^−^) for infected (LASV) and uninfected (control) mice.

To characterize the overall T-cell response during human LF, we examined the presence of virus-specific T cells and antigenically unrelated T cells during the acute phase of infection, as well as shortly after discharge from the Lassa ward. Using major histocompatibility complex (MHC) class I tetramers bound to the HLA-A2-restricted LLGTFTWTL LASV peptide (8), we observed that the generation of epitope-specific CD8 T cells was limited during the acute phase of the disease but increased significantly after patient discharge (Fig. 2A). However, evaluation of the activated-T-cell compartment, defined as CD38^+^ or CD38^+^ HLA-DR^+^ cells (24, 25), indicated the opposite trend, with a significantly higher percentage of activated T cells observed during the acute phase than during the postrecovery phase (Fig. 2B). LASV-specific CD8 T cells also expressed activation markers during acute infection, but the overall frequency of activated cells (up to 22% of total CD8 T cells) (Fig. 2B) indicated polyclonal CD8 T-cell activation that could include LASV-specific and -nonspecific T cells. Previous studies in HIV patients identified antigenically unrelated CD8 T cells expressing high levels of CD38 (26). Following this criterion, we utilized Epstein-Barr virus (EBV)-specific MHC class I tetramers to determine whether antigenically unrelated T cells were also activated during acute LF. We observed a discrete subset of EBV-specific CD8 T cells that expressed CD38 during LASV infection but not in healthy controls, suggesting that non-LASV-specific T-cell activation indeed occurred during acute Lassa fever (Fig. 2C).

To further determine whether nonspecific T-cell activation occurred during LASV infection *in vivo*, we utilized a mouse model of severe infection previously developed in our laboratory that allows functional studies of T cells during LF (6). We engineered a mixed bone marrow chimera using recipient type I interferon (IFN) receptor knockout (IFNAR^−/−^) mice transplanted at a 1:1 ratio with bone marrow progenitor cells from wild-type (CD45.1) mice and TCR-transgenic mice designed to recognize the MHC class I-restricted ovalbumin (OVA) peptide SIINFEKL (herein referred to as OT-I mice) (Fig. 2D). To track the activation of bystander T cells in this model, we searched for OT-I-specific CD8^+^ CD44^+^ T cells as previously described (11). Infection of these mixed chimeras with LASV via intranasal (i.n.) inoculation resulted in strong activation of the polyclonal wild-type T-cell repertoire in which roughly 90% of cells in blood expressed CD44 9 days after infection. The majority of this activated subset also downregulated the expression of the lymphoid tissue homing marker CD62L, suggesting recruitment of activated T cells into inflamed peripheral tissues (Fig. 2E and F). Although to a lesser extent, around 40% of OT-I T cells also upregulated CD44, indicating nonspecific-T-cell activation (Fig. 2F). In this case, 25% of the total OT-I CD8 T-cell pool also downregulated CD62L, indicating the presence of a subset of bystander T cells with the capacity for recruitment to LASV-infected tissues.

Taken together, our results indicated that antigenically unrelated T-cell activation takes place during LF in humans, as well as in a mouse model of infection. In the latter, our data suggest that a subset of bystander cells have the capacity to infiltrate LASV-infected tissues during infection.

### Severe LF is characterized by poor LASV-specific T-cell responses.

**FIG 3:**
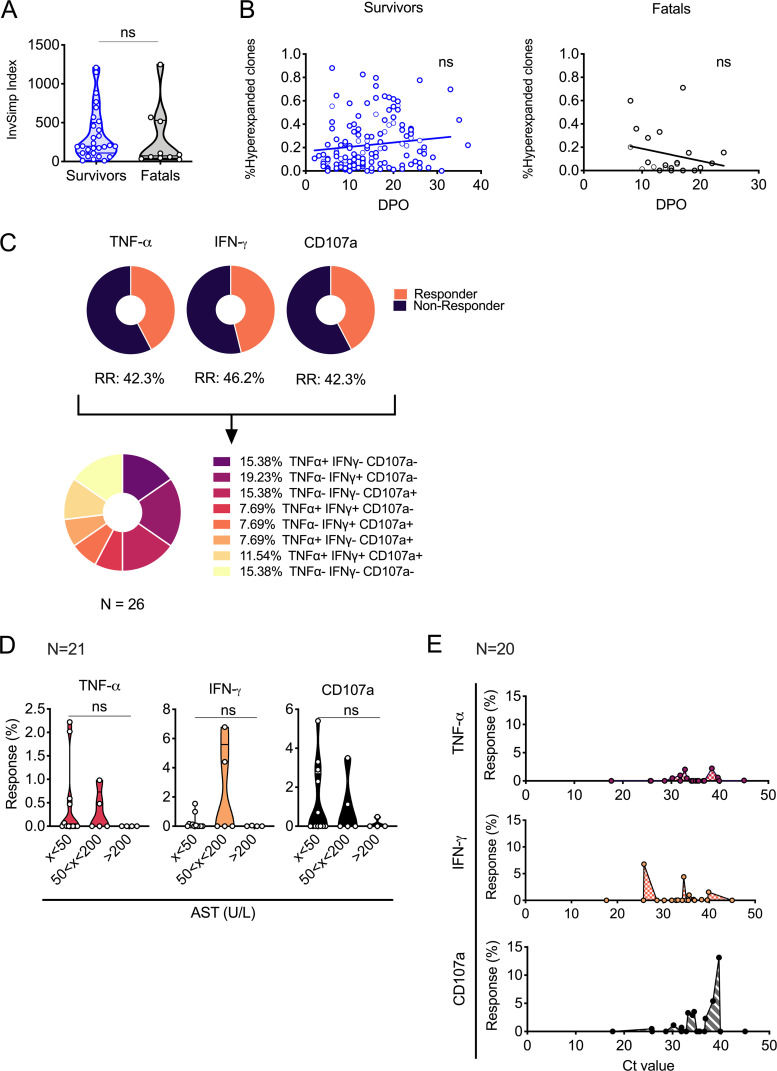
Clonal repertoires and effector T-cell responses in Lassa fever (LF) patients. (A) Cross-sectional T-cell repertoire diversity in LF survivors (*n* = 28) and fatal cases (*n* = 10) was analyzed by TCR sequencing and is represented as the inverse of Simpson’s index (InvSimp). Violin plots depict individual samples, sample distributions, median values, and quartiles. Statistical significance was determined using the Mann-Whitney test, and significance levels are presented as follows: ns, *P* > 0.05 (not significant); *, *P* ≤ 0.05; **, *P* ≤ 0.01; ***, *P* ≤ 0.001; ****, *P* ≤ 0.0001. (B) Longitudinal evaluation of hyperexpanded clones (see clonal space definitions in Materials and Methods) in *n* = 120 samples collected from survivors and *n* = 22 samples collected from nonsurviving patients. Individual samples and trend lines are depicted. ns, not significant (Spearman’s correlation analysis); DPO, days postonset. (C) Pie chart depicting the percentages of patient samples (*n* = 26) that showed effector response capacity as indicated by production of cytokines interferon gamma (IFN-γ) and tumor necrosis factor alpha (TNF-α) and degranulation marker CD107a/LAMP-1 in response to peptide stimulation. (D) Graphs representing cytokine responses of CD8 T cells in LF patients stimulated with Lassa virus (LASV) peptide pools. Cytokine responses and degranulation are reported across ranges of patient levels of serum aspartate transaminase (AST) (U/liter) (*n* = 21). Violin plots depict individual samples, sample distributions, median values, and quartiles. ns, not significant (Kruskal-Wallis followed by Dunn’s). (E) Cytokine responses and degranulation are reported across cycle threshold (*C_T_*) values as a readout of patient viremia (*n* = 20). Results for individual samples are depicted.

To better understand the dynamics of T-cell activation during acute LF, we sought to investigate the clonality and kinetics of the T-cell responses in patients during their stays at the Lassa ward. To this end, we performed TCR sequencing in peripheral blood samples of surviving and nonsurviving patients from the onset of symptoms to death or recovery. Evaluation of T-cell repertoire diversity showed no significant differences between fatalities and survivors in the acute phase of the disease, suggesting that similar levels of clonal T-cell expansion occur in fatal and nonfatal LF (Fig. 3A). However, we detected differences in the kinetic trends of the clonal T-cell expansion between survivors and fatalities. In survivors, the frequencies of hyperexpanded T-cell clones, that is, clones that dominate the overall T-cell response, increased toward the end of the patient’s stay at the ward in coincidence with virus clearance and discharge. Conversely, nonsurvivors showed the opposite trend, with hyperexpanded clones dominating the response at early time points after onset (Fig. 3B). These results suggested that, in fatal LF, the early antiviral response was dominated by T-cell clones with poor ability to clear virus.

One of the consequences of nonspecific T-cell activation is that it leads to a regulatory environment that can prevent the immune system from mounting an adequate response (19). Thus, we hypothesized that, in severe LF, the presence of antigenically unrelated cells could lead to diminished LASV-specific responses. To test this hypothesis, we evaluated the LASV-specific effector T-cell profiles in samples from patients showing different degrees of disease severity as measured by viremia and AST levels in blood. We utilized an LASV nucleoprotein (NP)-derived peptide pool to restimulate peripheral blood T cells and assessed their capacity to degranulate (by expression of surface CD107a) and produce effector cytokines like tumor necrosis factor alpha (TNF-α) and gamma interferon (IFN-γ). Within a sample size of *n* = 26 samples collected from *n* = 13 patients, 11.5% of samples showed a multifunctional T-cell response characterized by degranulation and production of both cytokines. However, we observed that in 15.4% of samples, T cells did not respond at all to restimulation with LASV antigen (Fig. 3C). In order to stratify these data across different ranges of disease severity, we evaluated whether there was a relationship between T-cell effector functions and biomarkers of severity like the levels of serum AST and viremia. We observed that, in samples with corresponding serum AST levels greater than 200 U/liter, T cells did not show any effector functions; namely, they neither degranulated nor produced any cytokines. IFN-γ production was greater in T cells from samples with corresponding AST levels between 50 and 200 U/liter, while TNF-α production was observable in that same range or lower (Fig. 3D). Similarly, T-cell effector functions were observable only in samples with linked low levels of viremia, namely, high cycle threshold (*C_T_*) values, indicating an association between high virus loads, liver pathology, and poor T-cell function (Fig. 3E).

These findings suggested that severe LF was associated with the expansion of T-cell clones with poor effector functions, resulting in lower capacity to control virus replication.

### Fatal LF is characterized by T-cell homing to inflamed tissues.

**FIG 4:**
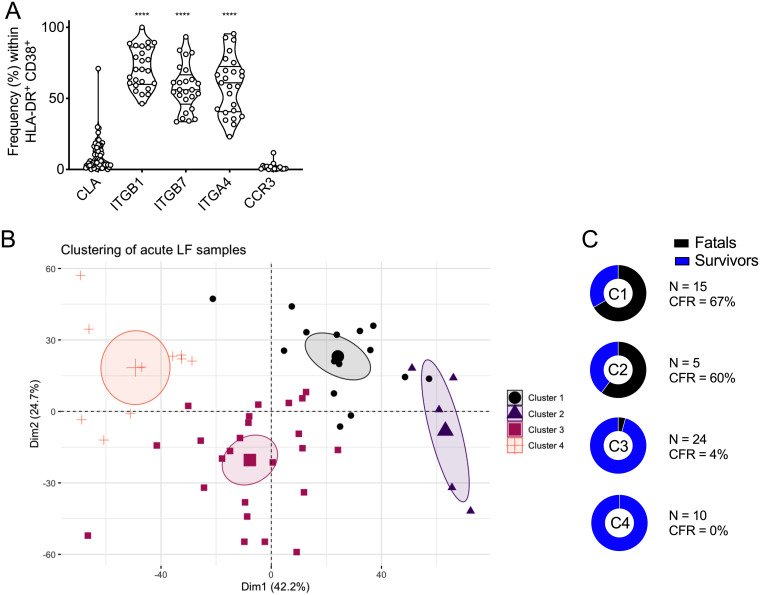
T-cell homing in human LF. (A) Freshly isolated patient PBMCs were analyzed by flow cytometry, and activated (CD38^+^ HLA-DR^+^) CD8 T cells from LF patients were analyzed for expression of the T-cell homing factors ITGB7, ITGA4, ITGB1 (*n* = 25), CCR3 (*n* = 16), and CLA (*n* = 61). Violin plots depict individual samples, sample distributions, median values, and quartiles. Statistical significance was determined using the Kruskal-Wallis test followed by Dunn’s posttest, and significance levels are presented as follows: ns, *P* > 0.05 (not significant); *, *P* ≤ 0.05; **, *P* ≤ 0.01; ***, *P* ≤ 0.001; ****, *P* ≤ 0.0001. (B) Principal-component analysis (PCA) of T-cell homing markers in the activated compartment (CD38^+^ HLA-DR^+^) of CD4 and CD8 T cells in cryopreserved patient samples (*n* = 54). Clustering of samples was based on hierarchical analysis to identify main subsets. (C) CFR for each cluster was calculated based on outcome of disease of the corresponding patient linked to each sample. Dim, dimension; C, cluster; CFR, case fatality ratio.

The identification of nonspecific T cells and poorly functional effector T cells in severe LF prompted us to evaluate whether these cells had the capacity to migrate to inflamed tissues. Thus, we investigated T-cell homing patterns in activated T cells during acute LF and their relationship with disease severity. In a first screening in Nigeria, we evaluated the presence of homing markers in the activated T-cell compartment of *n* = 36 patients. We evaluated the presence of CLA as a marker of skin homing (*n* = 61 samples), integrin-β1 (ITGB1), and ITGA4 (*n* = 25 samples) as general markers of lymphocyte homing to inflamed tissues, ITGB7 (*n* = 25 samples), which together with ITGA4, forms LPAM-1, driving T-cell homing to the gut, and CCR3 (*n* = 16 samples) as a homing marker for the respiratory mucosa (16, 21, 27). The data obtained in this screening indicated that during acute LF, the activated T-cell compartment was dominated by cells with homing capacity to inflamed tissues, the gut mucosa, and to a lesser extent, the skin and respiratory tract (Fig. 4A). To gain further insight into the relationship between T-cell homing and LF severity, we performed additional immunophenotyping of activated CD8 and CD4 T cells in cryopreserved samples from acute LF cases using multiparametric flow cytometry panels (*n* = 22 patients, *n* = 54 samples). We evaluated the levels of expression of markers of T-cell homing to skin, mucosae, and lymphoid tissue in activated T cells and determined sample clustering via principal-component analysis (PCA). We observed the presence of four defined clusters characterized by differences in disease outcome. Samples from fatal Lassa fever cases were predominantly found in clusters 1 and 2 (67% and 60% case fatality ratio [CFR], respectively), while samples from survivors formed the majorities grouped in clusters 3 and 4 (4% and 0% CFR, respectively) (Fig. 4B and C). These results suggested an association between T-cell homing signatures and LF outcome.

Overall, our data indicated that the acute phase of LF was characterized by activated T cells with homing capacity to inflamed tissues. These lymphocyte homing signatures drive patient clustering in groups with marked differences in disease severity.

### Unique T-cell populations in LF patients with different degrees of severity.

**FIG 5:**
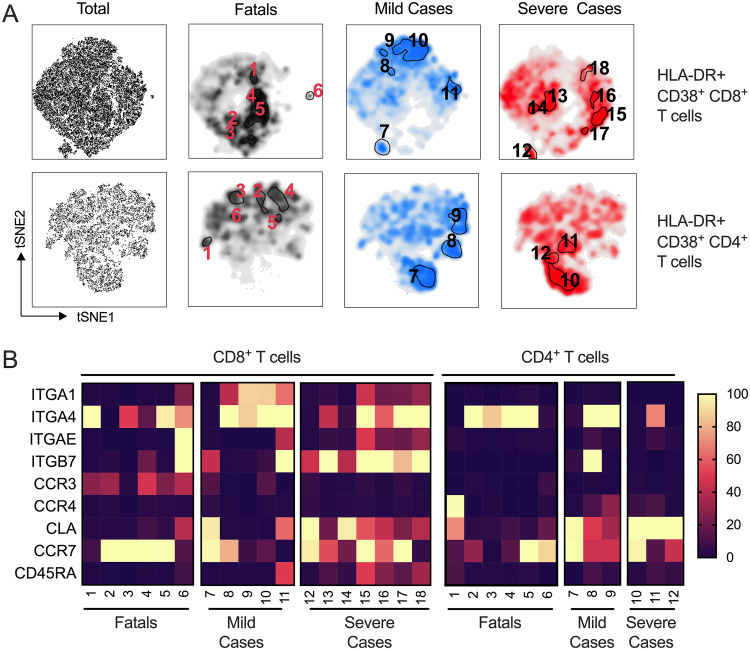
T-cell populations in LF patients. (A) Activated (CD38^+^ HLA-DR^+^) CD8^+^ and CD4^+^ T cells were concatenated across all patient samples (*n* = 54). *t*-Distributed stochastic neighboring embedding (*t*-SNE) was calculated on the expression of homing markers CLA, CCR3, CCR4, CCR7, ITGA4, ITGA1, ITGAE, and ITGB7 and expression of CD45RA. Outcome-based population analysis was performed by automated density-based gating. Numbers indicate T-cell subsets identified in the t-SNE analysis. (B) For each identified population, the percentage (%) of contribution from each outcome was verified, and the expression frequency (%) of each marker is shown according to the color key on the right. CCR, chemokine receptor; CLA, cutaneous lymphocyte antigen; ITG, integrin.

To better understand the relationship between T-cell homing and LF severity, we next sought to dissect the homing signatures of the polyclonal T-cell response in severe, mild, and fatal LF. Due to the fact that T-cell-mediated immunity is polyclonal, the overall response reflects the combined action of different T-cell subsets with overlapping and nonoverlapping functions. We reasoned that, within the polyclonal repertoire that characterized the activated T-cell compartment during acute LF, there might be virus-specific and nonspecific T cells with different homing signatures. Thus, we utilized multidimensional flow cytometry followed by *t*-distributed stochastic neighbor embedding (t-SNE) analysis to investigate the presence of unique T-cell subsets and their homing signatures in fatal, mild, and severe LF cases (Fig. 5A). Samples from fatal LF cases were enriched in activated CD8 and CD4 T-cell subsets with a central memory phenotype (CCR7^+^ CD45RA^−^), suggesting, perhaps, activation of T-cell clones not specific for LASV. Clones expressing high levels of ITGA4 were also dominant in both the CD4 and the CD8 compartment. In the latter, we also observed clones that expressed CCR3, which suggested migration of CD8 T cells to the respiratory tract (Fig. 5B). Among survivors, severe cases showed a CD8 T-cell clonal space that was dominated by CD45RA^+^ effector T cells that either retained CCR7 expression or not. Also, severe cases showed the highest presence of CD8 T-cell clones with homing capability to the gut (ITGA4^+^ ITGB7^+^). Interestingly, samples from this cohort also harbored clones with high expression levels of CLA in both the CD8 and CD4 T-cell compartments. These results indicated that severe LF is characterized by effector T cells capable of migrating to the gut and skin. Finally, samples from mild cases that maintained low levels of viremia and serum AST throughout the acute phase of disease showed low expression levels of CCR7 in most of the T-cell subsets and overall low levels of CD45RA, consistent with an effector memory phenotype. In mild cases, we observed clones with strong expression of ITGA4, suggesting migration to sites of inflammation, but overall low levels of tissue-specific homing factors in CD8 T cells. We further observed gut and skin signatures in CD4 T cells.

Taken together, our findings suggested that fatal LF is associated with reactivation of central memory T-cell clones with the capacity to migrate to sites of inflammation like the respiratory mucosa. Both severe and mild cases showed the presence of effector T cells with the capacity for homing to the gut and skin and revealed differences in homing signatures between CD8 and CD4 T cells.

### Mucosal exposure to LASV results in high lethality in a mouse model.

**FIG 6:**
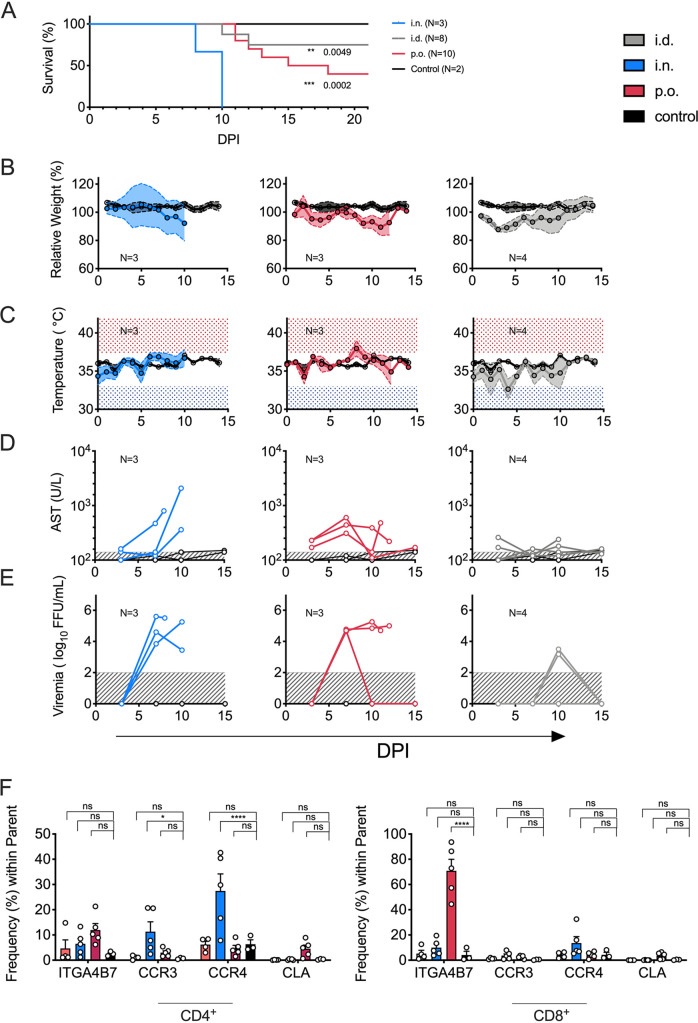
Route-dependent LF severity in mice. (A) IFNAR^B6^ chimeras were infected with 1,000 FFU of LASV either i.n., p.o., or i.d. Uninfected mice served as the control. Survival is shown in Kaplan-Meier curves. Statistical evaluation was performed via Mantel-Cox test. DPI, days postinfection. (B to E) Longitudinal analysis of morbidity parameters in IFNAR^B6^ chimeras infected with LASV via different routes, including relative weight loss (B), fluctuations in body temperature (C), levels of serum aspartate transaminase (AST) (D), and viremia (E). FFU, focus-forming units. (F) Frequencies of homing markers in peripheral blood cells were analyzed for each infection route 9 days postinfection by flow cytometry on effector (CD44^+^ CD62L^−^) CD4 and CD8 T cells. For data shown in panels B to F, statistical significance was determined using two-way ANOVA, followed by Dunnett’s multiple-comparison test. Across the figure, significance levels are presented as follows: ns, *P* > 0.05 (not significant); *, *P* ≤ 0.05; **, *P* ≤ 0.01; ***, *P* ≤ 0.001; ****, *P* ≤ 0.0001. In panel C, shaded areas highlight body temperature extremes due to fever (red, >37.4°C; gray, <33°C). In panels D and E, shaded areas (gray) mark limits of detection. i.n., intranasal; i.d., intradermal; p.o., oral. Throughout the figure, inoculation routes are color coded as shown next to panel A.

Our findings in patients strongly suggested that fatal and severe LF were associated with the migration of activated T cells, particularly CD8 T cells, to infection sites such as the gut and respiratory mucosae. Because T-cell homing is imprinted by antigen-presenting cells located primarily at the site where T cells first encounter antigen (23, 28, 29), we reasoned that the observed differences in disease severity could be related to some extent to the route of infection. To test this hypothesis, we utilized IFNAR^B6^ bone marrow chimeras (IFNAR^−/−^ mice reconstituted with wild-type [CD45.1] bone marrow), in which we previously showed that intraperitoneal infection of LASV resulted in 100% lethality (6). To test the effect of the inoculation route in this model, we inoculated mice with 1,000 focus-forming units (FFU) of LASV via intranasal (i.n.), intradermal (i.d), or oral (p.o.) inoculation. Intranasal infection of IFNAR^B6^ chimeras with LASV resulted in 100% lethality and the shortest time to death after infection (8 to 10 days). Oral administration of LASV also resulted in high lethality (60%) in this model, even though the time to death was delayed to nearly 20 days postinfection in some subjects. Conversely, intradermal inoculation of LASV resulted in 25% lethality (Fig. 6A). Furthermore, mice that were inoculated either intranasally or orally with LASV lost weight over the course of the disease and had fever, high levels of circulating AST, and high levels of viremia. Intradermal inoculation resulted in a milder disease phenotype characterized by low serum AST, the absence of fever, and low levels of viremia (Fig. 6B to E).

In agreement with the findings in LF patients, the severities of disease after inoculation by intranasal and oral routes in the mouse model were correlated with increases in the frequencies of circulating T cells with homing signatures for the respiratory mucosa (CCR3 and CCR4) and the gut (ITGA4B7) (Fig. 6F), which suggests that the identification of circulating T cells with defined homing markers during acute LF may provide useful information about routes of infection. These data, together with the observations in human LF cases, strongly suggest that mucosal exposure to LASV is associated with severe disease and high lethality.

## DISCUSSION

In this study, we present clinical immunology data collected in LF patients treated at the ISTH Lassa ward during the Nigerian LF outbreak of 2018. There are several clades of LASV circulating in Nigeria, and clade distribution is closely related to geography. Since all patients came from Edo and its neighboring states, it is highly likely that all patients were infected with viruses of lineage II (30). Still, a possible confounding factor in our study could be the association of specific viruses with pathogenesis, although this would be more likely at the sublineage level.

With this caveat in mind, our research was focused on trying to understand the role of T cells in LF pathogenesis. Previous work has highlighted the potential role of T-cell-mediated immunopathology of LASV infection in animal models (6, 7). However, there is much less information about whether T-cell-mediated immunopathology may influence severe and fatal LF in humans.

We observed that, during the acute phase of LF, there was substantial activation of T cells that were antigenically unrelated to LASV. In particular, we demonstrated that EBV-specific CD8 T cells were activated during acute LF in humans and that OVA-specific T cells were also activated during LASV infection in a mouse model. The first finding could be due to activation of bystander T cells and/or antigen-specific activation of CD8 T cells due to EBV reactivation. Reactivation of latent virus infections, including EBV and cytomegalovirus (CMV), has been previously reported as a consequence of alterations of immune homeostasis leading to inflammation and immune suppression (14, 26) and has been observed in severe viral infections like Ebola virus disease (14) and influenza (31). The OT-I T-cell activation observed in the mixed bone marrow chimera mouse model, however, can only be due to bystander T-cell activation, since the stimulating antigen (OVA) was never present. This phenomenon is common to many viral infections or autoimmune disorders. In the context of viral infection, bystander T-cell activation may result in either enhanced immunity or enhanced immunopathology (26, 31, 32). Although our data are not quantitative, there are some indications that nonspecific T-cell activation may be an important component of LF immunopathology. We have observed that in a mouse model of infection, bystander T cells generated after LASV infection have the capacity to migrate to inflamed tissues. There is substantial evidence that recruitment of bystander T cells to mucosal tissues can cause tissue damage (15, 32, 33). Our findings in the mouse model are also in agreement with the *t*-SNE analysis of T cells from human fatalities, which showed homing capacity of T cells with a central memory phenotype. In addition, we speculate that the activation of T cells antigenically unrelated to LASV may be correlated with the hyperexpansion of T-cell clones during the early time points after disease onset in fatal LF. Since fatal LF cases sustained high levels of viremia throughout the course of disease, it is conceivable that a fatal outcome may be attributed in part to the expansion of nonspecific T-cell clones that dominate the response and are unable to control virus replication. For example, activation of antigenically unrelated T cells has been shown to delay recruitment of virus-specific T cells to the lungs in mouse models of respiratory virus infections (34), and bystander T cells formed during virus infection have been shown to enhance virus-induced autoimmune responses (35). An additional consequence of bystander T-cell formation is conversion of effector CD4 T cells into Foxp3^+^ peripheral regulatory T cells (Tregs). This has been shown previously to promote a regulatory environment in which antigen-specific T-cell immune responses are diminished (19). In that regard, we have observed that LASV-specific effector T-cell responses are remarkably diminished in severe and fatal LF, with a direct association between elevated viremia and serum aminotransferases and poor T-cell functionality. The poor LASV-specific T-cell effector responses observed in severe LF are also in agreement with previously published *in vitro* data in which direct infection of DCs led to poor T-cell activation (36). Although we do not know whether LASV primarily infects antigen-presenting cells in humans, we observed a correlation between high viral loads and poor formation of LASV-specific effector T cells.

In an IFNAR^B6^ chimeric mouse model with a functional hematopoietic system, inoculation of LASV via different routes resulted in significantly different degrees of disease severity. This finding was in agreement with the association between severe LF and lymphocyte homing to gut and respiratory mucosa observed in humans. Interestingly, in these IFNAR chimera mice, mucosal exposure (oral and intranasal) resulted in remarkably higher lethality than skin exposure. Although we do not have exposure data in humans, previous studies have demonstrated that, as opposed to Ebola virus disease, LF is caused by multiple spillover events from rodents into the human population, rather than by human-to-human transmission (5, 37). Epidemiological studies performed during previous LF outbreaks suggested that risk factors for infection with LASV include activities that may involve contact with rodent excreta, such as consumption of contaminated food or inhalation of dust particles harboring virus particles (3, 4, 38). Skin contact with rodents via bites or while preparing rodents for food have also been reported (2, 39). It would be worthwhile to further investigate whether mucosal exposure to LASV leads to a more severe phenotype than skin exposure. These experiments could also serve to determine whether lymphocyte homing signatures could provide information about routes of transmission and, therefore, disease severity, which has the potential for significant public health relevance.

Finally, our data suggest that in severe LF, T-cell activation does not necessarily lead to efficient LASV-specific T-cell responses and virus control in humans but, rather, results in enhanced immunopathology and disease severity. This is in agreement with previous findings in an LASV-susceptible mouse model (6). In addition, similar studies conducted in mice infected with lymphocytic choriomeningitis virus (LCMV), the prototypic Old World arenavirus, have underscored a dual role of CD8 T cells during infection. Whereas CD8 T cells are essentially required for early control of LCMV replication, they can also worsen immune-mediated pathology in more severe models of infection (40). These results are therefore in line with our findings in human LF. Although counterintuitive, we propose that it could be worthwhile to test the effect of postexposure therapies aimed at depleting T cells in acute LF, at least in preclinical models like nonhuman primates and perhaps in combination with antivirals.

## MATERIALS AND METHODS

### Ethics statement.

The present study was performed with approval of the Irrua Specialist Teaching Hospital (ISTH) Research and Ethics Committee (ISTH/HREC/20171208/43) and the Ethics Committee of the Medical Association of the State of Hamburg (PV3186).

### Patients and samples.

All samples were obtained in the period between January 2017 and June 2018 from acute LASV patients that were diagnosed and treated at Irrua Specialist Teaching Hospital in Edo state, Nigeria. Patients were enrolled in the study at admission to the Lassa ward at ISTH. Samples were collected from patients every 2 days from the day of admission until they were discharged. All patients received ribavirin treatment upon their admission to the ward. Lassa diagnostics and viral loads were determined in EDTA–whole-blood samples using the commercial RT-PCR Lassa kit 1.0 (Altona Diagnostics). For evaluation of clinical chemistry parameters, including AST levels, plasma was analyzed using a SpotChem machine.

### Isolation of PBMCs and HLA typing.

Human peripheral blood mononuclear cells (PBMCs) were isolated from whole-blood samples using Lymphoprep gradient medium and SepMate technology. PBMCs were either directly used for analysis or cryopreserved in 90% heat-inactivated fetal calf serum (FCS) and 10% dimethyl sulfoxide (DMSO) for posterior analysis. For selection of HLA-A2^+^ patient samples, DNA was extracted using the DNeasy blood and tissue kit (Qiagen) according to the manufacturer’s instructions. DNA was used for PCR-based genotyping using an HLA-A low-resolution screening kit (Olerup).

### Phenotyping of cells with flow cytometry.

Cells were washed with phosphate-buffered saline (PBS) prior to staining. Cryopreserved human PBMCs were thawed in prewarmed RPMI 1640 medium containing 10% human serum (HS), 1× streptomycin, 1× penicillin, 1× l-glutamine and in the presence of DNase I. Cell viability was determined using a live/dead dye staining method for 20 min (1/1,000 dilution) (Zombie dye; BioLegend). Fc-γ receptor blocking was performed with either murine or human BD Fc block (BD). After blocking, cells were stained with antibodies against extracellular antigens with an antibody cocktail diluted in PBS containing 5% FCS. After that, cells were fixed and permeabilized with Cytofix/Cytoperm (BD) for 45 min and then stained with antibodies against intracellular antigens if required (Table 1). Samples were acquired using either a benchtop Guava cytometer (Millipore) (Nigeria) or an LSR Fortessa cytometer (BD) (Hamburg). Data analysis was done with FlowJo software (Tree Star). Cell population gating was adjusted with fluorescence-minus-one (FMO) controls. Viability was assessed in relation to corresponding AST serum levels (U/liter) by linear regression.

**TABLE 1:** Antibodies utilized for flow cytometry^*a*^

Reactivity	Antibody	Conjugated fluorophore^*b*^	Clone	Company
Antihuman	Integrin-α1/CD49a	APC	TS2/7	BioLegend
Antihuman	Integrin-α4/CD49d	PE/Dazzle 594	9F10	BioLegend
Antihuman	Integrin-α4/CD49d	APC	9F10	BioLegend
Antihuman	Integrin-αE/CD103	BV711	Ber-ACT8	BD Biosciences
Antihuman	Integrin-αE/CD103	BUV395	Ber-ACT8	BD Biosciences
Antimouse	CD103	PerCP/Cy5.5	2E7	BioLegend
Antihuman	CD14	APC/Cy7	HCD14	BioLegend
Antihuman	CD16	APC/Cy7	3G	BioLegend
Antihuman	CD19	Alexa Fluor 700	HIB19	BioLegend
Antihuman	CD193 (CCR3)	APC/Cy7	Clone5E8	BioLegend
Antihuman	CD193 (CCR3)	PerCP/Cy5.5	Clone5E8	BioLegend
Antihuman	CD193 (CCR3)	PE	Clone5E8	BioLegend
Antimouse	CD193 (CCR3)	APC Fire 750	J073E5	BioLegend
Antihuman	CD194 (CCR4)	BV605	L291H4	BioLegend
Antimouse	CD194 (CCR4)	PE/Cy7	2G12	BioLegend
Antihuman	CD195 (CCR5)	BV421	HEK/1/85a	BioLegend
Antihuman	CD197 (CCR7)	BV421	G043H7	BioLegend
Antihuman	CD197 (CCR7)	BV711	G043H7	BioLegend
Antihuman	CD3	BV421	OKT3	BioLegend
Antihuman	CD3	BV510	OKT3	BioLegend
Antimouse	CD3	FITC	17A2	BioLegend
Antihuman	CD38	PerCP/Cy5.5	HIT2	BioLegend
Antihuman	CD38	BV510	HIT2	BioLegend
Antimouse	CD3ε	PE/Dazzle 594	145-2C11	BioLegend
Antihuman	CD4	PerCP/CY5.5	SK3	BD Biosciences
Antihuman	CD4	BUV737	SK3	BD Biosciences
Antihuman	CD4	BV711	SK3	BioLegend
Antimouse	CD4	BUV737	GK1.5	BD Biosciences
Antimouse	CD44	BV395	IM7	BD Biosciences
Antimouse	CD45.2	PE	104	eBioscience
Antimouse	CD45.1	APC	A20	BioLegend
Antimouse	CD45.1	FITC	A20	BioLegend
Antimouse	CD45.2	Alexa Fluor 700	104	BioLegend
Antihuman	CD45RA	BV711	HI100	BioLegend
Antihuman	CD56	APC/Cy7	HCD56	BioLegend
Antihuman	CD56	BV510	HCD56	BioLegend
Antihuman	CD56 (NCAM)	Alexa Fluor 700	5.1H11	BioLegend
Antimouse	CD62L	BV785	MEL-14	BioLegend
Antihuman	CD8a	FITC	RPA-T8	BioLegend
Antihuman	CD8a	BV510	RPA-T8	BioLegend
Antihuman	CD8a	PE/CY7	RPA-T8	BioLegend
Antihuman	CD8a	BV650	RPA-T8	BioLegend
Antimouse	CD8a	BV650	53-6.7	BioLegend
Antihuman/mouse	CLA	PE	HECA-452	BD Pharmingen
Antihuman	CLA	FITC	HECA-452	BioLegend
Antihuman	HLA-DR	PE/Cy7	L243	BioLegend
Antihuman	HLA-DR	PerCP/Cy5.5	L243	BioLegend
Antihuman	HLA-DR	FITC	L243	BioLegend
Antihuman	IFN-γ	APC	4S.B3	BioLegend
Antihuman	Integrin-β1/CD29	PE/Cy7	TS2/16	BioLegend
Antihuman	Integrin-β1/CD29	Alexa fluor 700	TS2/16	BioLegend
Antihuman/mouse	Integrin-β7	PE	FIB27	BioLegend
Antihuman/mouse	Integrin-β7	PerCP/Cy5.5	FIB27	BioLegend
Antihuman	LAMP-1/CD107a	PE/CY7	H4A3	BioLegend
Antimouse	LPAM-1/α4β7 integrin	BV421	DATK32	BD Biosciences
Antihuman	TNF-α	BV785	MAb11	BioLegend
Antihuman	TNF-α	PE	MAb11	BioLegend

a The table depicts all antibodies utilized in flow cytometry for characterization of T-cell phenotypes and functional profiles.

b APC, allophycocyanin; PE, phycoerythrin; PerCP, peridinin chlorophyll protein; FITC, fluorescein isothiocyanate.

For identification of epitope-specific CD8 T cells, we utilized staining with HLA-A*02:01-based tetramers conjugated with previously validated LASV and EBV peptides. Tetramers were generated using a custom Flex-T MHC tetramer kit (BioLegend) using signal peptides according to the manufacturer’s instructions. Tetramers were labeled with phycoerythrin (PE) and allophycocyanin (APC) fluorochromes.

### High-dimensional analysis of flow cytometry data.

Multiparametric analysis of data was performed in R statistical software using the gplots, mclust, and factoextra packages. Clustering was performed by using ward.D2 and a Euclidian-distance-based algorithm. Principal-component analysis was performed on the correlation matrix. Cluster number was chosen manually based on hierarchical clustering of samples by hclust function.

For *t*-SNE analyses, the populations of activated T cells (CD8 and CD4, separately) were selected and concatenated across all samples for each disease category, respectively, using FlowJo software. These pools were then downsized based on the least-abundant population and again merged to generate one forward-scatter (FSC) file on which *t*-SNE analysis was performed using the *t*-SNE plugin. Populations were gated by automated density selection. The contributions of cells originating from the three outcome pools and the expression frequencies of homing markers were identified for each population.

### TCR repertoire analysis.

TCR sequences were obtained by amplifying the TCR β CDR3 loci from PBMC RNA. The TCR library was generated via multiplex PCR using 45 forward primers, each specific to a functional TCR Vβ segment, and 13 reverse primers, each specific to a TCR Jβ segment. Forward and reverse primers were pooled separately to a final stock of 10 μM each. Multiplex PCR amplification was performed in a first step with the use of a 1:5 mix of primers without and with overhang adapters, which serve as the specific target of primers in a second PCR amplification. The Qiagen multiplex PCR kit was used at a final volume of 25 μl, including 1 μl of DNA template and 2.5 μl of Q solution. The PCR cycle was initiated by a 15-min step for activation of the HotStartTaq DNA polymerase at 95°C. Next, denaturation at 94°C for 30 s was followed by annealing for 90 s at 69°C, with touchdown PCR reduction of the temperature by 1°C for the first 10 cycles. After extension at 72°C for 90 s, 40 cycles (10 cycles for touchdown plus 30 cycles) of denaturation, annealing, and extension followed. A final extension at 72°C was performed for 10 min. A second PCR step was performed to include Nextera XT dual indices and Illumina sequencing adapters. The Qiagen multiplex PCR was used once again in a final volume of 25 μl, including 5 μl of DNA template and 2.5 μl of each primer. The PCR cycle was initiated with an activation step of 15 min at 95°C followed by 15 cycles of denaturation for 30 s at 95°C, annealing for 30 s at 55°C, and extension for 30 s at 72°C. A final extension at 72°C was performed for 5 min. PCR products were purified after each PCR run with the use of Agencourt AMPure XP reagent and following the manufacturer’s instructions. PCR products were mixed with a 1.8× volume of beads for the recovery of amplicons of about 200 bp. The R package tcR was used for TCR data analysis. Simpson inverse diversity was calculated using the repDiversity function based on read proportions. Clonal expansion was calculated using the clonal.space.homeostasis function, defining clones as rare (0 < X < 1e−05), small (1e−05 < X < 1e−04), medium (1e−04 < X < 0.001), large (0.001 < X < 0.01), and hyperexpanded (0.01 < X < 1).

### T-cell stimulation assays.

PBMCs were resuspended in 10% HS, 1× streptomycin, 1× penicillin, 1× l-glutamine at a concentration of 2.5 × 10^6^ cells/ml. Cells were stimulated with an LASV-specific peptide pool at a concentration of 1 μg/ml for each individual peptide. All peptides were selected using the immune epitope database (IEDB) prediction tool utilizing an artificial neural network (ANN) prediction method for MHC class I binding prediction. Peptides were predicted for all common HLA-A and HLA-B alleles to cover >95% of the Nigerian population. Only peptides with a predicted 50% inhibitory concentration (IC_50_) of <100 nmol were selected. A peptide pool was generated comprising peptides found in both LASV strain Ba366 (accession number GU830839.1), which is a commonly used laboratory strain, and field-isolated strain Nig-08-A47 (Irrua, Nigeria, 2008) (accession number ADU56631.1) to increase the likelihood of sequence conservation. Phorbol myristate acetate (PMA)/ionomycin (50 ng/ml, 2.5 μM) was used as a positive stimulation control. PBMCs were incubated with the peptides for 6 h (37°C, 5% CO_2_). To prevent cytokine release into the medium, cells were treated with GolgiStop (BD) protein transport inhibitor (1/1,000 dilution) for the last 5 h. Plates were sealed airtight and placed at 4°C overnight and then stained for flow cytometric analysis. Viability was assessed in relation to corresponding AST serum levels (U/liter) by linear regression. After background subtraction, negative values were set equal to 0, and all responses above 0 were considered for each cytokine separately. Responses were considered multifunctional for the respective study sample if the response to peptide stimulus resulted in above-background responses for multiple cytokines within the respective CD8 T-cell population.

### Mice.

All mouse strains—C57BL/6, C57BL/6_Ly5.1 (B6.SJL-Ptprca Peb/BoyJ), IFNAR^−/−^ [B6(Cg)-Ifnar1tm1.2Ees/J], and OT-I [C57BL/6-Tg(TcraTcrb)1100Mjb/J]—were purchased from The Jackson Laboratory. All mouse colonies were maintained in the animal housing facility at BNITM, Hamburg. All animal experiments were conducted according to the guidelines of the German animal protection law and under approvals 31/17 and 92/18 issued by the state of Hamburg. Bone marrow chimeras were generated as previously described (41). Briefly, 6- to 10-week-old recipient mice were lethally irradiated (550 rad, 4 h apart, by a cesium source) and reconstituted with 3 × 10^6^ donor bone marrow cells.

### Experimental infection of mice.

Mice were infected with 1,000 FFU of LASV (recombinant Ba366 strain). Intranasal inoculation was performed under isoflurane anesthesia by application of 25 μl of virus diluted in PBS directly to the nostrils. Oral administration of virus was performed via application of a 100-μl inoculum with the aid of a sterile buttoned cannula (22 gauge), 3 cm in length and 1.25 mm in diameter. Intradermal inoculation of LASV was also performed under isoflurane anesthesia. Using an electric shaver, fur was shaved off in a 1-cm by 1-cm area on the back of the mouse just above the tail base. Hairs were brushed aside, and the skin was gently scratched using UV-sterilized sandpaper (P150 to P400 granularity). On the scratched surface, a 10-μl sterile inoculum was pipetted and distributed evenly. After application, mice were kept under anesthesia for 15 min.

Animals with severe signs of disease, such as temperature of <28°C or weight loss of >20%, were euthanized according to our approved protocol guidelines. For evaluation of clinical chemistry and viremia, 30 to 50 μl of blood was drawn by tail vein puncture every 3 to 7 days over the course of the experiment. Levels of AST in serum were analyzed with a commercial kit and using a Fujifilm machine. Sera were diluted 1:10 or higher with a 0.9% saline solution (vol/vol). The normal range of AST in bone marrow chimeric mice has been determined as <200 U/liter. Viremia and virus titers in organs were determined using plaque assays to determine the presence of focus-forming units as described elsewhere. When criteria for euthanasia were fulfilled or at the end of the experiment, animals were euthanized with an isoflurane overdose followed by cervical dislocation. Murine PBMCs were isolated from whole-blood samples collected at day 9 postinfection. Red blood cell lysis was performed using red blood cell lysis buffer (10×; BioLegend) according to the manufacturer’s instructions. PBMCs were directly analyzed through flow cytometry.

All staff carrying out animal experiments passed training programs according to category B or C of the Federation of European Laboratory Animal Science Associations.
